# Fertility preservation and monitoring in adult patients diagnosed with lymphoma: consensus-based practical recommendations by the Fondazione Italiana Linfomi & Società Italiana della Riproduzione Umana

**DOI:** 10.3389/fonc.2023.1252433

**Published:** 2023-09-12

**Authors:** Carla Minoia, Simonetta Viviani, Erica Silvestris, Simone Palini, Francesca Parissone, Giuseppe De Palma, Anna Fedina, Gennaro Cormio, Attilio Guarini, Guido Gini, Luigi Montano, Francesco Merli, Fedro Alessandro Peccatori

**Affiliations:** ^1^Hematology Unit, IRCCS Istituto Tumori “Giovanni Paolo II”, Bari, Italy; ^2^Division of Onco-Hematology, IEO, European Institute of Oncology IRCCS, Milan, Italy; ^3^Gynecologic Oncology Unit, IRCCS Istituto Tumori “Giovanni Paolo II”, Bari, Italy; ^4^Physiopathology of Reproduction Unit, Cervesi Hospital, Cattolica, Italy; ^5^Department of Obstetrics and Gynecology, Azienda Ospedaliera Universitaria Integrata di Verona, Università di Verona, Verona, Italy; ^6^Institutional BioBank, Experimental Oncology and Biobank Management Unit, IRCCS Istituto Tumori “Giovanni Paolo II”, Bari, Italy; ^7^Data Office Fondazione Italiana Linfomi, Alessandria, Italy; ^8^IRCCS Istituto Tumori Departiment of Interdisciplinary Medicine (DIM), University of Bari, Bari, Italy; ^9^Clinic of Hematology Azienda Ospedaliera Universitaria (AOU) delle Marche, Ancona, Italy; ^10^Andrology Unit and Service of Lifestyle Medicine in UroAndrology, Local Health Authority (ASL), Salerno, Italy; ^11^Hematology Unit, Azienda Unità Sanitaria Locale-IRCCS di Reggio Emilia, Reggio Emilia, Italy; ^12^Gynecologic Oncology Department, European Institute of Oncology IRCCS, Milano, Italy

**Keywords:** fertility preservation, lymphoma, chemotherapy, quality of life, survivorship, sexuality, long-term side effects, consensus-based reccommendations

## Abstract

**Introduction:**

Fertility preservation (FP) and monitoring has considerable relevance in the multidisciplinary approach to cancer patients. In these consensus-based practical recommendations, the scientific societies Fondazione Italiana Linfomi (FIL) and Società Italiana della Riproduzione Umana (SIRU) reviewed the main aspects and identified the optimal paths which aim to preserve and monitor fertility in patients diagnosed with lymphoma at the different phases of the disease and during long-term survivorship.

**Methods:**

For the Panel, eleven experts were selected for their expertise in research and clinical practice on onco-fertility and lymphoma. The Panel’s activity was supervised by a chairman. A series of rank-ordering key questions were proposed according to their clinical relevance and discussed among the Panel, focusing on patients diagnosed with non-Hodgkin’s lymphomas and Hodgkin lymphoma. Agreement among all the Panelists on the content and terminology of the statements was evaluated by a web-based questionnaire according to the Delphi methodology.

**Results:**

From the literature review a total of 78 questions or sentences, divided into the 6 areas of interest, were identified. By applying the Gwet's AC, k was: Section 1: 0,934 (Very good); Section 2: 0,958 (Very good); Section 3: 0,863 (Very good); Section 4: 0,649 (Good); Section 5: 0,936 (Very good); Section 6 raw agreement 100%. Two rounds of Delphi allowed to provide the maximum agreement. All statements were newly discussed in a round robin way and confirmed for the drafting of the final recommendations.

**Discussion:**

These recommendations would be useful for onco-hematologists, gynecologists, urologists, and general practice physicians who take care of young lymphoma patients to guarantee an evidence-based oncofertility assessment and treatment during the oncologic pathway.

## 1 Introduction

In 2020, around 627,439 people were diagnosed with lymphoma worldwide; in Europe there were 19,858 new cases of Hodgkin lymphoma (HL) and 86,321 of non-Hodgkin lymphomas (NHL) (1, 2). Among female patients aged 20 to 39 years, HL and NHL accounted for 3,304 and 2,639 new cases, whereas the incidence for male patients was aged 20-59 years was 6,696 and 20,733, respectively (1, 2). Long-term survival of patients diagnosed with lymphoma has significantly improved in the last decades and the population of long survivors has grown substantially (3–6). Among the complications following treatment for lymphoma, permanent loss of fertility in patients of childbearing age is a relevant and potentially quality of life-impairing effect of cancer treatment. In order to estimate the risk of fertility impairment, the following aspects are more relevant than others: patient sex and age, type and dose of chemotherapy regimen, site and dose of radiation therapy, and, in females, ovarian reserve (7–9).

In male patients, cytotoxic treatment targets rapidly dividing cells including spermatocytes, thus disrupting spermatogenesis and potentially leading to infertility after cancer treatment at any age. On the contrary, in females, chemo-and/or radiotherapy induce age-dependent ovarian damage, leading to oocyte depletion and premature ovarian insufficiency (POI). As younger female patients, i.e. those under the age of 30 years, have a higher number of follicles and might have a regular ovulatory cycle even with a small numbers of follicles, less severe gonadal damage is reported in younger compared to older patients, who may enter chemotherapy- induced POI more often (7).

Although over time attention has been paid to aspects of gonadotoxicity for young patients in order to reduce the use of drugs which lead to a risk of permanent infertility for HL and NHL, there are some patients in whom this risk cannot be reduced. An example is cases in which it is necessary to use intensified dose front-line regimens, or in which first-line therapy proves ineffective and a high-dose rescue regimen followed by autologous stem cell transplant (ASCT) is necessary (10). Furthermore, it is important to underline that long-term survivors currently face the late effects of chemotherapy regimens mainly used in the past, especially for the treatment of HL, which have historically demonstrated a significant rate of infertility. Among front line regimens for HL, those containing procarbazine are associated with a significant risk of gonadotoxicity. Some have been given in the past (i.e.MOPP: mechlorethamine, vincristine, procarbazine, prednisone; COPP: cyclophosphamide, vincristine, procarbazine, prednisone), while others are still being used nowadays (escalated BEACOPP: bleomycin, etoposide, doxorubicin, cyclophosphamide, vincristine, procarbazine, prednisone) (11, 12). Alkylating agents (mechlorethamine, melphalan, carmustine, lomustine, chlorambucil, busulfan, cyclophosphamide) can cause gonadotoxicity depending on the cumulative dose and age at administration (8, 13, 14). Among salvage regimens, those containing platinum and its derivatives are associated with gonadotoxicity (14, 15). For all these agents, it should be taken into account that, the cumulative risk of gonadal toxic effects are increased by their use in combination regimens and by the number of cycles administered. Recently, the targeted drugs (e.i. brentuximab vedotin, polatuzumab vedotin, checkpoint inhibitors, BCL-2 inhibitor venetoclax, Bruton’s tyrosine kinase inhibitors ibrutinib, zanubrutinib, acalabrutinib, pirtobrutinib and the PI3K inhibitors idelalisib and duvelisib) have been introduced in the clinical practice. Their gonadotoxicity is still unknown, since on the one hand they are mainly used in the relapsed/refractory setting in patients who have already received multiple lines of chemotherapy regimens, and on the other hand they can be administered for a prolonged number of cycles. Their gonadotoxic potential is therefore difficult to be assessed (14–16).

With regard to radiotherapy (RT), the use of more advanced technologies and PET (positron emission tomography)-oriented approaches has made it possible to limit the use of this technique to the abdominal and pelvic regions, progressively reducing the patients at risk. It is known that the testis is extremely sensitive to radiation. Radiation doses as low as 0,1–1,2 Gy can impair spermatogenesis, while doses higher than 4 Gy can cause permanent azoospermia. Gonadotoxicity can occur even if RT is delivered to pelvic nodes without testicular shielding at doses of ≥ 20 Gy, and when it is given concurrently with chemotherapy, doses of 9–10 Gy may induce gonadal dysfunction (7, 17, 18). RT may also induce severe injury to the ovarian reserve. In adult female patients, RT at doses > 6 Gy to the ovaries as well as the whole abdomen or the pelvic nodes will cause ovarian damage, and doses > 30 Gy will also affect uterine function with a raised incidence of spontaneous miscarriage and low fetal intrauterine growth (7, 8, 16, 17).

Considering the aspects presented above, it is of utmost importance to assist clinicians and patients in the recognition of the potential risk of infertility as a consequence of specific treatment modalities, and to provide and effectively implement onco-fertility counseling with an open discussion on the impact of lymphoma and its treatment on reproductive function (9, 19, 20). After histologic diagnosis, available fertility preservation (FP) options should be discussed prior to treatment start at the earliest possible opportunity. The expedited referral for interested patients to reproductive specialists should be facilitated in order to promptly evaluate the risks and benefits of the more appropriate PF procedures based on an accurate assessment of the individual risk of gonadotoxicity. It is also important to allow adequate time to complete FP in order to avoid a delay in the start of cancer treatment. With regard to the options available, it should be emphasized that these also depend on national legislation, in fact some procedures, such as the production and freezing of embryos before anti-cancer treatment, are not permitted in all countries.

The Fondazione Italiana Linfomi (FIL) “Survivorship, Quality of Life and Comorbidity Committee’’ and the Società Italiana della Riproduzione Umana (SIRU) collaborated to review the current evidence on this issue in adult patients with lymphoma and to develop a multidisciplinary consensus paper. Consensus was obtained among the expert Panel by applying the Delphi method. The document presents shared statements of practical help in the management of young patients with HL or NHL about to start therapy and for their short and long-term follow-up.

## 2 Methods

The multi-disciplinary working group for this consensus-based position paper, hereafter referred to as the Panel, comprised five onco-hematologists belonging to the Fondazione Italiana Linfomi (FIL) – “Survivorship, Quality of Life and Comorbidity Committee” (CM, SV, AG, GG, FM), four gynecologists or andrologists expert in fertility preservation and reproduction affiliated with the Società Italiana della Riproduzione Umana (SIRU) (ES, GC, FP, LM), and one embryologist and one biologist with expertise in tissue preservation (SP, GDP) affiliated with the SIRU. The experts were selected according to their expertise in research and clinical practice in lymphoma, FP, survivorship, and quality of life. The Panel was supervised by an international expert leader on onco- fertility (FAP) (chairman). Data management and analyses were conducted by the FIL data office in the person of AF. The project was carried out following the Declaration of Helsinki’s ethical principles for medical research involving human subjects, and in accordance with the Good Clinical Practice regulations.

The project was presented and discussed at the annual meeting of the FIL in November 2021. Following the experience gained in previous cooperative studies, the Panel designed a series of practical questions on the topic of fertility preservation and follow-up in patients diagnosed with and treated for lymphoma (16, 19). The series of rank-ordering key questions was proposed according to clinical relevance and discussed among the Panel. The questions were discussed and shared by the Panel during a preliminary online meeting in February 2022. The focus of the questions regarded patients diagnosed with NHL and HL, aged 18- 40 years for females and > 18 years for male patients. Specifically, the following histotypes of NHL were considered: Diffuse large B-cell lymphoma (DLBCL), primary mediastinal B-cell lymphoma (PMBL), Follicular lymphoma (FL), Peripheral T-cell lymphoma (PTCL), Mantle Cell Lymphoma (MCL) and Burkitt lymphoma (BL). Both classical and nodular lymphocyte-predominant HL were included.

Six main key topics were addressed: i) to which categories of patients is this document addressed?; ii) pre-therapy counseling for both female and male patients; iii) the optimal medical treatment for fertility preservation during chemotherapy for female patients; iv) the follow-up: indications on fertility tests to be carried out in the period following chemotherapy (1-5 years and > 5 years from remission); v) safe conception in both female and male patients; vi) disorders of the sexual sphere after anti-tumor therapy. **Table 1** summarizes the rank-ordering key topics discussed among the Panel.

**Table 1:** Rank-ordering key topics discussed among the Panel on fertility preservation in lymphoma patients.

**Q1- To which of patients in this document addressed?** Q1A- Patients diagnosed with non-Hodgkin’s lymphoma (NHL) Q1B- Patients diagnosed with lymphoma (HL) Q1C- Patients to be treated with pelvic node(s) radiotherapy
**Q2- Pre-therapy counseling** Q2A- Blood test suggested prior to/ during counseling Q2B- Specialist exams suggested during counseling Q2C- Preservation techniques Q2D- Definition of the timing for access to the counseling and preservation procedures ang their use
**Q3- Optimal medical treatment to be used in addition to or as the only method of preservation during chemotherapy treatment for female patients** Q3A- To which patients should gonadotropin-releasing homone agonist (GnRHa) administered? Are there any contraindications? Q3B- Up to what age to prescribe GNRHa?
**Q4- The follow-up period: indications on fertility test to be carried out in the period following chemotherapy (1-5 years and > 5 years from disease remission)** Q4A- Which tests should be performed in female lymphoma survivors and how often? Q4B- Which tests should be performed in male lymphoma survivor and how often?
**Q5- How long after the end of cancer treatments can conception be considered safe?**
**Q6- Assessment and treatment of the disorders of the secual sphere after anti-tumor therapy** Q6A- To whom to address the evaluation of disorders of the sexual sphere? Q6B- What tools to use to evaluate any alterations in the sexual sphere in the post-therapy phase

A qualitative literature review (using Mesh and free text terms) was conducted from January 1990 to January 2022, with no language restrictions. Five panelists (CM, SV, ES, SP, FP) conducted the literature review addressing the selected clinical key questions. For each key topic, the search was conducted by two independent reviewers (title and abstract selection, full text paper reading, data reporting) on the three main search engines: MEDLINE (via PubMed), the Cochrane Library, and EMBASE. The following types of articles were considered eligible: cohort studies, case-control studies, randomized clinical trials (RCT), systematic reviews, and meta-analyses. The search was carried out by combining the conditions (lymphoma, Hodgkin lymphoma, non-Hodgkin’s lymphoma, diffuse large B cell lymphoma, follicular lymphoma, mantle cell lymphoma, primary mediastinal lymphoma, Burkitt lymphoma, female patient, male patient), interventions (e.g., chemotherapy, immunotherapy, radiotherapy, GnRH analogs, GnRH antagonist, GnRH agonist, gonadorelin, leuprorelin, triptorelin, enantone, decapeptyl, buserelin, goserelin) and outcomes of interest (oocyte cryopreservation, ovarian tissue cryopreservation, semen cryopreservation,conception, pregnancy, post-treatment parenthood, post-treatment childhood, acute ovarian failure, premature ovarian insufficiency, primary ovarian insufficiency, premature ovarian failure, azoospermia, infertility, sterility,gonadotoxicity, follicle-stimulating hormone FSH, luteinizing hormone LH, estradiol E2, anti-Müllerian hormone AMH, testosterone TST, prolactin PRL, inhibin B, sexuality, counseling, follow-up). A revision of current oncologic, hematologic, and gynecologic guidelines was also carried out.

**Figure 1:**
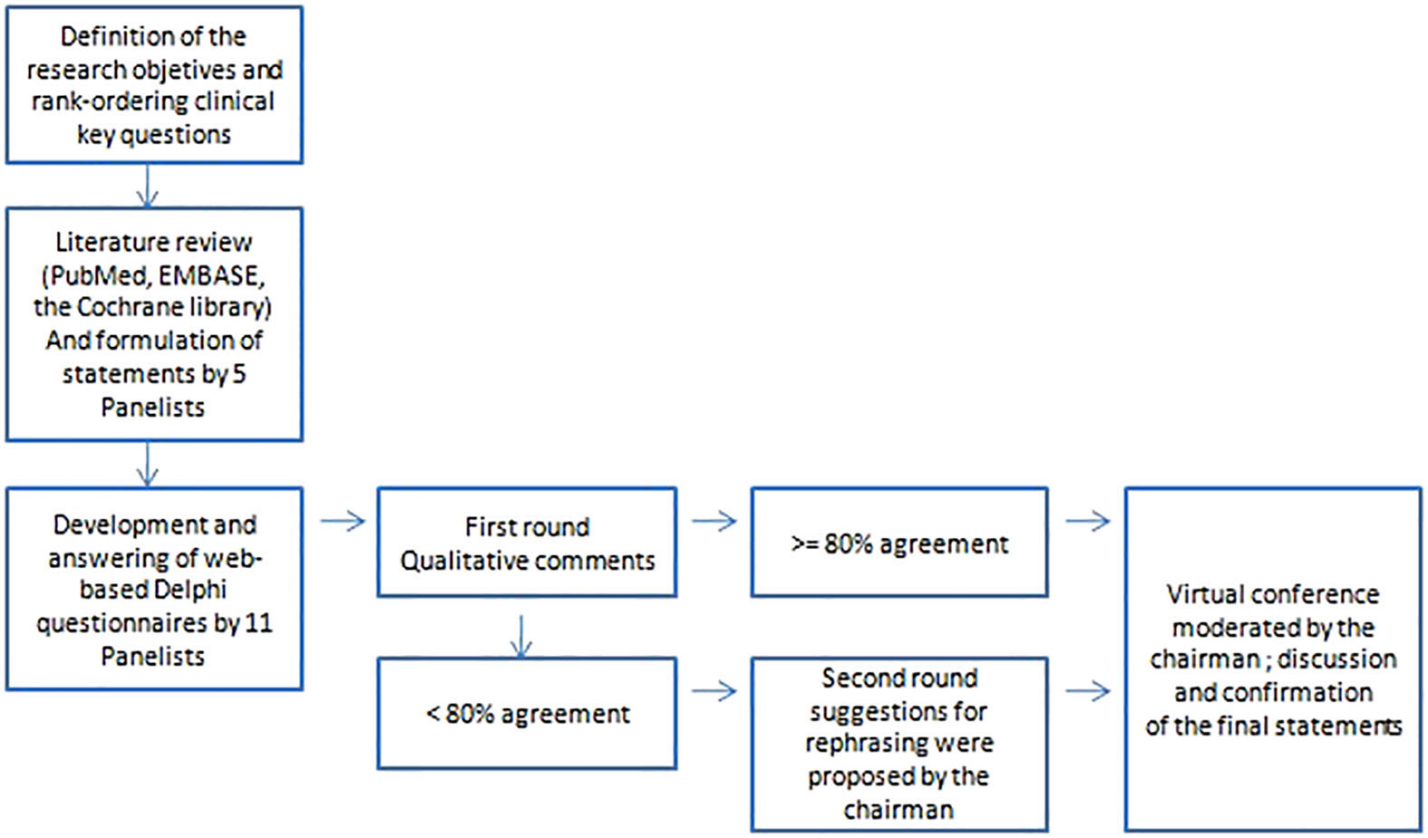
Diagram of the Delphi process used for the consensus-based statements.

The agreement among all the Panelists on the content and terminology of the statements was scored by a (web-based) questionnaire according to the Delphi methodology (21, 22). The methodology is represented in **Figure 1**. When a consensus for each question of at least ≥ 80% was not obtained, the statement was discussed and suggestions for rephrasing were proposed by the chairman. The inter-rater reliability has been calculated by Gwet’s agreement coefficient (AC). A kappa below 0.2 was indicative for poor agreement and a kappa above 0.8 for very good agreement. The strength of agreement is detailed as follows: kappa < 0.2: Poor; > 0.2 ≤ 0.4: Fair; > 0.4 ≤ 0.6: Moderate; > 0.6 ≤ 0.8: Good; > 0.8 ≤ 1: Very good.

A second round of votes as a web-based questionnaire was then performed for statements not reaching an agreement ≥ 80% (Very good).

At the end of this process, the Panel met in a virtual conference moderated by the chairman held in December 2022. All statements were newly discussed in a round robin way and confirmed for the drafting of the final recommendations. The scientific committees of Fondazione Italiana Linfomi (FIL) and Società Italiana della Riproduzione Umana (SIRU) have revised and approved this consensus paper.

The following outcomes define female infertility in the selected papers: a) Primary ovarian insufficiency (POI) defined as depletion or dysfunction of ovarian follicles with cessation of menses before age 40 years (previously been referred to as premature menopause or primary ovarian failure) (23); POI may comprise acute ovarian failure or premature menopause. Acute ovarian failure (AOF) is defined as the immediate loss of ovarian function after chemotherapy or radiation therapy, which may be transient, or as the permanent loss of ovarian function within 5 years of cancer diagnosis (24). Premature menopause (PM) is defined as the retention of ovarian function for at least five years following cancer diagnosis and the occurrence of non-surgical menopause before age 40 (25). b) Decreased ovarian reserve defined as the reduction in oocyte quantity (oocyte number) by measurements of hormone levels of AMH, FSH, E2 and by transvaginal ultrasonographic measure of the sum of the number of antral follicles (ACF) in both ovaries, and by a reduced response to ovarian stimulation compared with women of comparable age (26, 27) c) Infertility defined as the failure to achieve a successful pregnancy after ≥12 months of regular, unprotected sexual intercourseor due to an impairment of a person’s capacity to reproduce either as an individual or with her/his partner (28).

## 3 Results

From the literature review a total of 78 questions or sentences, divided into the 6 areas of interest, were identified. The 1^st^ topic “To which categories of patients is this document addressed?” consisted of 13 statements; the 2^nd^ topic “Pre-therapy counseling” of 2 statements; the 3^rd^ topic “Optimal medical treatment to be used in addition to or as the only method of preservation during chemotherapy treatment for female patients” of 3 statements; the 4^th^ topic “The follow-up period: indications on fertility tests to be carried out in the period following chemotherapy (1-5 years and > 5 years from disease remission)” of 35 statements; the 5^th^ topic “How long after the end of cancer treatments can conception be considered safe?” of 3 sentences; the 6^th^ topic “Assessment and treatment of the disorders of the sexual sphere after anti-tumor therapy” of 3 sentences The complete list of questions of Delphi round 1 is available in **Supplementary 1**.

In the first round of Delphi, conduced for the 78 statements, an agreement of ≥80% was obtained for 65 questions (83.3%), and a full agreement (100%) for 46 (58.9%) sentences. By applying the Gwet’s AC, k was: Section 1: 0,934 (Very good); Section 2: 0,958 (Very good); Section 3: 0,863 (Very good); Section 4: 0,649 (Good); Section 5: 0,936 (Very good); Section 6 raw agreement 100%. Data are summarized in **Table 2**.

**Table 2:** - Inter-rater Gwet's AC agreement for the 6 Sections of the consensus.

Section	items	% agreement	Gwet's AC
**1**	13	0,938	**0,934**
**2**	21	0,960	**0,958**
**3**	3	0,879	**0,863**
**4**	35	0,747	**0,649**
**5**	3	0,939	**0,936**
**6**	–	–	** -**

Raters n=11. Section 6 not calculated, ratings not varying.

For Section 4, the statements for which a ≥80% agreement had not been reached were rephrased into 13 additional sentences that made up round 2 of Delphi. At this point a full concordance was reached, with a 100% agreement in 94.4% of cases (**Supplementary 2**).

The following paragraphs concern the shared statements and the supporting literature. Main statements for the management of fertility preservation in young patients with HL or NHL from diagnosis through survivorship are summarized in **Table 3**.

**Table 3:** Statements of practical help in the management of fertility preservation in young patients with HL or NHL of childbearing age from diagnosis through survivorship.

Regardless of the risk associated with the specific chemotherapy drug or regimen, patients should receive fertility counseling for the choice of the optimal FP strategy before starting anti-cancer treatment. Hematologic Centers are advised to establish a multidisciplinary FP team with the nearest Fertility Center (and *vice versa*) in order to improve referral pathways, reduce time loss and optimize procedures; this will allow an expanded access to FP options.
The correct timing for onco-fertility counseling is as soon as possible, ideally at the time of diagnosis, in order to increase the patient’s awareness and to allow optimization of timing to apply FP techniques, especially in female patients. The aim is to offer onco-fertility counseling within 24-48 hours.
Pre-treatment evaluation for female patients: fertility counseling including pelvic US evaluation, AMH, FSH and LH on the 3rd-5th day of the menstruation; AMH and AFC are particularly useful for prediction of response to ovarian stimulation in case of mature oocyte cryopreservation procedure.
Pre-treatment evaluation for male patients: fertility counseling eventually including hormonal level measurement (FSH, LH, possibly inhibin B) at the time of semen cryopreservation.
Infective blood exams to be performed before gamete/tissue cryopreservation are: anti-HCV Ab, HBsAg, anti-HBcAb, anti-HIV and VDRL. They are optional for EU law, but mandatory in Italy.
FP techniques for female patients: - Mature oocyte cryopreservation should be proposed to patients in whom the delay of chemotherapy of 10-14 days is considered safe. Ovarian stimulation should be started as soon as possible (random start protocol) before chemotherapy. - Ovarian tissue cryopreservation (OTC) can be proposed as a unique technique in patients with: therapeutic urgency, a high/moderate gonadotoxic risk (>50%), no contraindication for surgery. The ovarian samples should be analyzed in order to exclude the presence of neoplastic cells by using molecular and histological analyses prior to graft, especially for aggressive NHL histotypes. The use of GnRH analogue during chemotherapy is recommended as an adjunct and not as an alternative to in vitro fertilization and cryopreservation of oocytes and ovarian tissue for FP.
Post-pubertal males should be offered sperm cryopreservation prior to the administration of gonadotoxic therapies. Surgical sperm extraction by conventional testicular biopsy (TESE) or microdissection testicular sperm extraction (micro-TESE) is an alternative strategy in the case of failure of the former.
Follow-up after anti-cancer therapy: - females: history of menses, FSH, estradiol, AMH, and AFC count by transvaginal US evaluation. The tests should be performed after an interval of at least one year following chemotherapy completion, as this is the suggested interval before attempting pregnancy in order to reduce the risk of pregnancy complications. - males: semen analysis, collected after 2–7 days of abstinence, recording sperm count, morphology, vitality and motility. The first assessment should be performed not prior to 12 months from the end of anti-cancer therapy. Serum FSH, inhibin B and inhibin B to FSH ratio may be used as surrogate markers of impaired spermatogenesis in patients who decline or are not able to perform semen analysis.
Disorders of the sexual sphere could be investigated during the follow-up by The Female Sexual Function Index (FSFI) and The International Index of Erectile Function (IIEF)

FP, fertility preservation; US, ultrasound; AMH, Anti-Müllerian Hormone; FSH, Follicle-Stimulating Hormone; LH, Luteinizing Hormone; AFC, Antral Follicle Count.

### 3.1 To which categories of patients is this document addressed?

The Panel intended to focus the present document on patients diagnosed with NHL and HL, aged 18- 40 years for females and > 18 years for male patients. Specifically, the following histotypes of NHL were considered: Diffuse large B-cell lymphoma (DLBCL), primary mediastinal diffuse B-cell lymphoma (PM-DLCL), Follicular lymphoma (FL), Peripheral T-cell lymphoma, Mantle Cell Lymphoma (MCL), and Burkitt lymphoma (BL). Both classical and nodular lymphocyte-predominant HL were included.

It is important to note that the majority of studies available on risk of gonadotoxicity after lymphoma treatment are retrospective and related to small patient population, moreover they use different outcomes, in particular for female patients, and different periods of follow-up after the end of treatment.

Chemotherapy-induced gonadotoxicity is influenced by the type of agent (e.g. alkylating agents), the dose intensity, and in women also by age at diagnosis. We can distinguish regimens with a high risk of infertility (≥ 80%), an intermediate risk (40-60%), a low risk (<20%), and of unknown risk (15, 16, 29). Regardless of the risk associated with the specific drug or regimen, patients should receive fertility counseling for the choice of the optimal FP strategy before starting anti-cancer treatment. This concept should be underlined because, especially for diseases with unfavorable prognostic factors at the onset, it could be necessary to initiate a second line therapy and ASCT in the case of an inadequate response.

This document is also addressed to patients who have completed the treatment courses for lymphoma, in order to guide the monitoring of the reproductive sphere.

#### 3.1.1 Patients diagnosed with non-Hodgkin’s lymphoma

R-CHOP chemotherapy is considered the gold standard treatment for the majority of patients diagnosed with DLBCL, the most frequent histotype among NHL. R-CHOP given for no more than 6 cycles in females younger than 40 years is associated with a low risk of gonadal dysfunction (<20%) (30, 31). The rate of POI and amenorrhea due to R-CHOP could reach 40-60% (intermediate risk) in female patients treated over 35 years of age (32). The majority of male patients treated with 4-6 cycles of CHOP/R-CHOP will recover spermatogenesis within 2 years from end of treatment, and permanent azoospermia has been documented in about 10% of men (33).

An intermediate risk of infertility (40-60%) is reported also after the intensified regimens used for the treatment of specific histotypes of aggressive NHL: DA-EPOCH-R (dose-adjusted etoposide, prednisone, vincristine, cyclophosphamide, doxorubicin, and rituximab) and Hyper-CVAD (cyclophosphamide, vincristine, doxorubicin, dexamethasone, high-doses methotrexate and cytarabine) (34, 35). In these studies, amenorrhea had been the outcome.

High-dose conditioning therapies and ASCT represents a salvage strategy for eligible NHL and a first-line consolidation for some NHL histotypes. It is known that ASCT is associated with a high risk of infertility both in males and females. High-dose chemotherapy appears to be incompatible with a full recovery of spermatogenesis and causes POI and amenorrhea in more than 70% of female patients (10, 36). The most frequent conditioning regimen associated with infertility risk is BEAM (BCNU/carmustine, etoposide, cytarabine, melphalan).

In the setting of both aggressive and indolent NHL, the immunomodulatory agent lenalidomide is used within various lines of therapy. A pregnancy prevention program is routinely applied for this drug, considering its teratogenic risk (37).

In some settings of indolent NHL, patients could be started with anti-CD20 monoclonal antibody monotherapy (rituximab or obinutuzumab). Combining the experiences of rheumatic disorders and lymphomas, data are in favor of avoiding pregnancies within the 12 months after the last administration of rituximab, both for male and female patients (38, 39). There is no evidence on the gonadotoxic potential of obinutuzumab monotherapy in the recorded clinical trials, but it is reasonable to apply the same indications as for rituximab. There is no data about the infertility risk of idelalisib, the PI3K inhibitor approved for relapsed/refractory FL, as well as of the targeted drugs polatuzumab vedotin, the BCL-2 inhibitor venetoclax, and the Bruton’s tyrosine kinase inhibitors ibrutinib, zanubrutinib, acalabrutinib and pirtobrutinib (40).

Regarding the use of CAR-T for the treatment of different histotypes of relapsed or refractory NHL, there is not a homogeneous approach regarding counseling for PF (41).

#### 3.1.2 Patients diagnosed with Hodgkin lymphoma

Poli-chemotherapy ABVD (adriamycin, bleomycin, vinblastine, dacarbazine), the regimen mainly used in the front-line treatment of HL, has a variable risk of infertility according to patient gender and age at administration. ABVD administered to male patients at any age and to female patients treated under the age of 35 years presents a low risk of infertility (< 20%), irrespective of the number of courses (42, 43). Available studies measure the restoration of menses but also the rate of live births (44). It should be emphasized that, when administered to female patients aged over 35 years, the risk of infertility increased and could reach 20-40% (42, 44,–45). In fact, ovarian function reserve measured by recovery of AMH one year after the end of 6 cycles of ABVD/AVD was significantly reduced compared with females younger than 35 years (46).

The intensified dose regimen escalated BEACOPP, which can be employed for the treatment of advanced stage or unfavorable risk classic HL, is generally associated with a high risk of infertility (> 70%) both in men and in women (47–50). The more cycles administered, the higher the risk of oligospermia/azoospermia, amenorrhea and POI (50, 51).

Some patients diagnosed with HL could benefit from treatment with targeted or innovative anti-tumor drugs (e.g. brentuximab vedotin, nivolumab, pembrolizumab). Even in the absence of clinical data, it is suggested to avoid conception for at least 6 months after brentuximab vedotin discontinuation both for male and female patients (40, 52). No clinical data are available on the gonadotoxic profile of the anti-programmed cell death 1 (PD-1) immune checkpoint inhibitor nivolumab and pembrolizumab, considering that the majority of patients who have access to these drugs are heavily pre-treated (53, 54).

#### 3.1.3 Patients to be treated with pelvic node(s) radiotherapy

Radiotherapy on subdiaphragmatic and pelvic regions is less and less used at fertile age. In anticipation of this treatment, generally performed as a consolidation after chemotherapy, fertility counseling is advised (17, 18, 32).

### 3.2 Pre-therapy counseling

#### 3.2.1 Blood tests suggested prior to/during counseling

For female patients, AMH (Anti-Müllerian Hormone) is the more appropriate hormonal ovarian reserve marker; FSH and LH (Luteinizing Hormone) may be additionally useful only if performed on the 3rd-5th day of the menstrual cycle. The usefulness of these tests is intended as a baseline value for the post-treatment ovarian reserve follow-up; if available, they can also be useful during the onco-fertility counseling together with other clinical information (e.g. age, previous obstetric history, AFC/Antral Follicle Count, proposed treatment). AMH, together with AFC, is also recommended for prediction of response to ovarian stimulation in case of oocyte cryopreservation procedure (55, 56). FP procedures should not be delayed if the hormonal balance exams are not yet available.

Infective blood exams to be performed before gamete/tissue cryopreservation are: anti-HCV Ab, HBsAg, anti-HBcAb, anti-HIV and VDRL. They are optional for EU law, but mandatory in Italy (57).

#### 3.2.2 Specialist exams suggested during counseling

Pelvic ultrasound examination with AFC performed by a gynecologic expert in Reproductive Medicine is recommended before/at the time of fertility counseling; this examination could be performed at any time of the menstrual cycle, but better if performed in the early follicular menstrual phase (58). The aim of this ultrasonographic evaluation is to exclude gynecological co-morbidity (such as ovarian cysts, endometriosis) and to assess the ovarian reserve.

For male patients no specialist exams are requested before cryopreservation. Counseling all males about the reproductive risks of cancer treatment and availability of FP options prior to initiation of cancer therapy and consideration of referral to a reproductive urologist is recommended. Although not mandatory, the assessment of hormonal level measurement (FSH, LH, inhibin B) at the time of semen cryopreservation in order to monitor the spermatogenesis restoration has also been suggested (59).

#### 3.2.3 Preservation techniques

Oocyte cryopreservation is a well-established FP technique that should be proposed to patients after personalized onco-fertility counseling, as is the possibility to delay treatment of 10-14 days, required to induce the multiple follicular growth. Ovarian stimulation and oocyte cryopreservation should be performed before starting chemotherapy (55, 56, 59–62). Some clinical conditions do not allow the patient to postpone the start of chemotherapy for 10-14 days and are characterized by therapeutic urgency: symptomatic and rapidly progressive disease, bulky mediastinal adenopathies at risk of superior vena cava syndrome, severe thrombocytopenia, disseminated intravascular coagulation, active infectious disease. In these cases, a medical approach with gonadotropin-releasing hormone agonist (GnRHa) following the FP counseling can be considered.

Ovarian tissue cryopreservation (OTC) has recently been considered a non-experimental technique by ASRM (60), but success and safety data are still limited. OTC should be proposed to patients diagnosed with lymphoma and no evidence of pelvic involvement at diagnosis (9, 55, 56). OTC could be proposed as a unique technique in patients with: therapeutic urgency, when chemotherapy has to be started within 10-14 days, when there is a high/moderate gonadotoxic risk, and if the patient’s clinical conditions are feasible for surgery. There is some literature data about OTC performed after a first-line low-gonadotoxic treatment exposure suggesting that it could be proposed in this situation after accurate counseling (55–62). OTC can be combined to both oocyte cryopreservation and ovarian transposition. In these cases, OTC should be performed immediately before starting ovarian stimulation and at the same time as ovarian transposition (9, 55–62).

To improve the safety of ovarian tissue transplantation (OTT) for patients in complete and prolonged survival after lymphoma, the ovarian samples have to be analyzed in order to exclude the presence of neoplastic cells by using molecular and histological analyses prior to graft, especially for aggressive NHL histotypes. Thus, an individual multidisciplinary evaluation of each case is required (55–62). The ovarian cortical strips may be grafted onto orthotopic sites, such as the atrophic ovary or pelvic peritoneum, close to infundibulopelvic ligaments or ovarian fossa, allowing the recovery of ovarian function and spontaneous pregnancy, or in heterotopic sites, such as the subcutaneous space of the forearm or abdominal wall, allowing recovery of endocrine function. So far, no recurrences due to OTT have been reported. Patients requiring pelvic radiation may also benefit from transposition of the ovaries to sites away from maximal radiation exposure (9, 55, 56, 62). Ovarian transposition can be performed at the same time as OTC.

Post-pubertal males should be offered sperm cryopreservation as this is the standard fertility-preservation method (62). Semen collection should be performed prior to the administration of gonadotoxic therapies such as chemotherapy or radiation therapy. It is also important to recognize that men with cancer may have underlying impairment in semen parameters prior to the administration of any oncologic therapy (50). Several factors associated with cancer can negatively impact male reproductive potential favoring azoospermia, including disruption of the normal hypothalamic-pituitary-gonadal axis and injury to the germinal epithelium as a result ofthe cytotoxic immune response to cancer, fever, and malnutrition (9, 33). This issue has been documented in the majority of patients with systemic symptoms of lymphoma (fever, night sweats, weight loss). For young men or for men who are unable to ejaculate, the following therapeutic options can be considered to obtain ejaculated sperm for cryopreservation: use of phosphodiesterase type 5 (PDE-5) inhibitors, vibratory stimulation, electroejaculation, and retrograde ejaculation.

Surgical sperm extraction by testicular biopsy (TESE) or microsurgical TESE (Micro-TESE) is an alternative strategy for males who cannot ejaculate via the aforementioned techniques, or who have azoospermia or insufficient sperm in the ejaculate (63). Testicular tissue cryopreservation is currently the only possibility to preserve fertility potential in children because sperm is not produced until puberty (64). Nonetheless, this technique remains experimental. Other techniques, such as *in vitro* culture or autologous transplantation of testicular tissue or precursor cells, are still being investigated, and in the future could be alternative strategies for FP. During the counseling, it is important to explain to the patients that the fertility potential of cryopreserved semen as well as semen from testicular biopsy decreases after cryopreservation (42).

### 3.3 Definition of the timing for access to counseling, preservation procedures and their use

The correct timing for onco-fertility counseling is as soon as possible, ideally at time of diagnosis, in order to increase the patient’s awareness and to allow optimization of timing to apply FP techniques (9, 61, 62). An urgent referral pathway needs to be established between the Hematological and The Reproductive Centers with the aim to offer onco-fertility counseling within 24-48 hours (55–62).

Counseling with an expert in Reproductive Medicine should be offered to all patients who desire to know the implications of the disease and its therapy on their reproductive potential; even when application of FP techniques is not possible or the patient is not interested in future childbearing, knowledge about gonadotoxic risk, future implications on fertility, fertility treatments in case of future infertility and other childbearing and parenting options (e.g./adoptions, oocyte/semen donation) could be of interest (55–62).

In order to optimize treatment and time, blood exams (see Q2) such as AMH and infective blood exams should be performed as soon as possible. These exams could be requested by the onco-hematologist prior to the onco-fertility counseling.

Ovarian stimulation should be started as soon as possible. If necessary, a random start protocol for ovarian stimulation is possible in order to minimize delay to oncologic treatments (55, 56, 61, 65). A prompt start to ovarian stimulation could also allow a double stimulation in case of poor ovarian reserve and the possibility of postponing the start of oncologic treatment (61, 62).

Laparoscopy for OT harvesting (OTC) should be performed as soon as clinical assessment of suitability for surgery is available. Usually from the moment of the decision, the organization of the procedure will take 24-48 hours.

Hematologic Centers are advised to establish a multidisciplinary FP team with the nearest Fertility Center (and vice versa) in order to improve referral pathways, reduce time loss and optimize procedures; this will allow an expanded access to FP options (9, 55–62).

### 3.4 Optimal medical treatment to be used in addition to or as the only method of preservation during chemotherapy treatment for female patients

#### 3.4.1 To which patients should gonadotropin-releasing hormone agonist be administered? Are there any contraindications?

The available evidence on the efficacy and safety of administering gonadotropin-releasing hormone agonist (GnRHa) during chemotherapy as an approach for ovarian protection in premenopausal oncologic patients with pathologies other than breast cancer is still limited. In fact, only four small randomized trials have involved women with hematologic malignancies (66). Among the several available meta-analyses, the largest one that groups the results from 3 randomized trials on hemato-oncological diseases included 109 patients diagnosed with HL and NHL (67). The median age for patients receiving chemotherapy regimens with different gonadotoxicity ranging from low (e.g. ABVD) to high (e.g. conditioning regimens for hematopoietic stem cell transplantation) was around 25 years. No significant difference in POI rates was observed between HL patients who received chemotherapy with or without concomitant GnRHa (18.9% vs. 32.1%; OR 0.70, 95% CI 0.20- 2.47), as well as for post-treatment pregnancies (17 vs. 18) (67). In one trial, which was included in the meta-analysis, AMH serum levels were assessed before and after treatment, with significantly higher levels of AMH in patients receiving GnRHa during chemotherapy at one year follow-up (68). Considering the available data and the good tolerability profile, the use of GnRH analogues during chemotherapy could be recommended as an adjunct and not as an alternative to *in vitro* fertilization and cryopreservation of embryos, oocytes and ovarian tissue for FP (69, 70). GnRHa should be administered every four weeks, starting preferably at least one week before the initiation of the first chemotherapy cycle and should be continued until after the administration of the last chemotherapy cycle (71). All patients who are candidates to receive GnRHa should be informed of the uncertainties regarding the potential role of GnRHa and the association with adverse events like hot flushes, bone and muscle pains, mood changes, and vaginal dryness (69, 70). GnRHa should not be considered as an alternative option to FP with cryopreservation techniques except for women for whom the latter is contraindicated due to treatment start delay or safety issues (55, 56).

Although it has been shown not to be effective as a FP method, hormonal estroprogestinic contraceptives are adopted by 12% of Italian hematologic centers affiliated with the FIL in conjunction with chemotherapy. In the same Italian surveys, the use of GnRHa during chemotherapy was used by 72.5% of the FIL hematologic centers (19).

#### 3.4.2 Up to what age should GNRHa be prescribed?

In order to prevent chemotherapy-induced POI and early menopause-related symptoms, the administration of GnRHa during chemotherapy is the only medical approach available for clinical use (55, 56, 71, 72). The mechanisms underlying the protective role of ovarian suppression with GnRHa during gonadotoxic treatments have not been fully understood (73). However, given the fast-acting effects and the suppressive ovarian function of GnRHa, these drugs may be able to protect the ovarian reserve from toxicity in adolescent girls and pre-menopausal women with ages between 15 and 45 years undergoing chemotherapeutic treatments (74).

### 3.5 The follow-up period: indications on fertility tests to be carried out in the period following chemotherapy (1-5 years and > 5 years from disease remission)

Evaluation of ovarian or testicular function after the end of cancer treatment and during the follow-up is not yet routinely performed in Italian hematological and oncological centers (19). While cardiac, pulmonary, and thyroid function as well as the occurrence of second cancers are closely monitored after lymphoma treatment, and several cancer survivorship guidelines recommend appropriate tests and timing of evaluation according to the known toxicities of the administered treatment, gonadal function is not part of these assessments, even though infertility, sexual hormone deficiency and sexual dysfunction have a significant impact on the quality of life of lymphoma survivors (75–78).

#### 3.5.1 Which tests should be performed in female lymphoma survivors and how often?

The assessment of gonadal function after lymphoma treatment in females includes history of menses, hormonal evaluation of FSH, estradiol and AMH, and transvaginal ovarian ultrasound examination with AFC (62). Asking the patient about the presence or absence of spontaneous regular menses is the first and simplest method to evaluate ongoing ovarian activity. Transient amenorrhea, in particular if lasting longer than 12 months from the end of chemotherapy, has been demonstrated to be a risk factor for subsequent infertility (62, 79). However, regular menses cycles after chemotherapy have been reported also in infertile females, those who were not able to remain pregnant, due to reduced ovarian reserve, and those who developed POI (79).

FSH cut-offs varied among the studies due to the use of different FSH assays and reagents (80). A value of FSH serum concentration greater than 25 UI/L, alongside low estradiol, is the most established diagnostic test for POI (46, 81). Although simple to perform, it is not useful in the diagnosis of poor ovarian reserve until high thresholds are used. In fact, normal FSH levels have been reported in patients with compromised ovarian reserve precluding FSH levels as a good predictive marker of the chance of conception (82, 83). Furthermore, large inter-cycle variations in basal FSH levels have been reported, and although the appropriate timing of FSH measurement should be on day 3 of the menstrual cycle, this timing may be difficult in females with irregular periods of menses after chemotherapy (84). Measurement of cycle day 3 estradiol levels as well as FSH/LH ratio and inhibin-B levels seems not to be useful in the prediction of ovarian reserve (85).

In contrast, serum levels of AMH, which is produced by granulosa cells of the recruited growing follicles in the ovary, were found to be a reliable marker of ovarian reserve and predictive for POI (56, 86, 87). In patients treated for Hodgkin lymphoma, AMH serum levels decreased during chemotherapy, more profoundly after BEACOPP than after ABVD, with a reciprocal increase of FSH (79, 81, 88). After the end of chemotherapy, AMH recovery to pre-treatment baseline values was observed within one year in ABVD-treated patients, whereas in BEACOPP-treated patients, AMH levels remained significantly lower than at baseline (89, 90). Study results suggest that pre-treatment AMH may predict mid- and long-term ovarian function. In female survivors with restored menstrual cycles, AMH recovery may be highly variable. Pregnancy can occur even in the presence of low post-chemotherapy AMH levels, below 1 ng/mL, documenting that AMH levels do not predict short-term fertility, unless associated with amenorrhea (91, 92).

Transvaginal ultrasound analysis is very useful to determine ovarian volume and, most importantly, AFC has been proposed as a surrogate marker for ovarian reserve evaluation (42). The performance of AFC only for predicting failure to achieve pregnancy is poor, mainly because AFC determines the number of oocytes but not the oocyte quality, on which pregnancy outcome also depends (91). Nowadays, no available test may help predicting pregnancy or live birth reliably in women undergoing anticancer therapy. The combination of AMH levels and AFC may be more accurate than a single exam in predicting ovarian reserve and menstrual function, as stated by Loverro G et al. in a small cohort of 29 female patients treated for HL (sensitivity of 83% and specificity of 88%), but this issue deserve further research (92).

Recovery of ovarian function can occur from several months to years after treatment completion and therefore there is no agreement between experts on the optimal time interval between end of lymphoma treatment and first gonadal function assessment, frequency and duration of surveillance. Gonadal function should be tested by measurement of AMH and AFC at the request of the patient or when pregnancy is desired. AMH below 0.5-1.1 ng/ml and AFC 5–7 follicles or less are suggestive for reduced ovarian function (23, 80, 93). The tests should be performed after an interval of at least one year following chemotherapy completion (94–97). In relation to the parameters found during the follow-up evaluation, an Assisted Reproductive Technology (ART) program could be suggested to the patient and the clinical decision would concern the proposal of using autologous or donor gametes in order to achieve maternity (98).

#### 3.5.2 Which tests should be performed in male lymphoma survivors and how often?

Testicular function should be evaluated at the request of the patient or when paternity is desired. Despite the 75-day duration of the spermatogenesis cycle, it is reasonable to suggest that the first assessment after potential gonadotoxic treatment should be performed not prior to 12 months from the end of therapy. This derives from the results of most of the studies which qualitatively and quantitatively evaluated spermatogenesis starting from 12 months after chemotherapy treatment (81, 99).

The simplest and most reliable method of assessing the effect of treatment on spermatogenesis is semen analysis, collected after 2–7 days of abstinence, recording sperm count, morphology, vitality and motility (7, 100). According to WHO reference limits, the normal sperm concentration is ≥ 15 x 10 (6) spermatozoa/mL, normal forms should account for at least 4%, and normal total motility (progressive and non-progressive) should be 40% (101).

Evaluation of gonadal function in male patients may include a physical examination and hormonal evaluation. Physical examination will be performed by the measurement of testicular size using scrotal ultrasonography or Prader orchidometer, which may reveal a decrease in testicular volume due to impaired sperm production and loss of tubular space (7, 102). Among hormonal evaluation, serum FSH, inhibin B and inhibin B to FSH ratio may be used as surrogate markers of impaired spermatogenesis in patients who decline or are not able to perform semen analysis. High levels of pituitary FSH indicate testicular dysfunction (79, 103). A cut-off level of 10.4 IU/L has been identified to predict azoospermia with specificity 81% (95% CI 76%-86%) and sensitivity 83% (95% CI 76%-89%) in childhood cancer survivors (104, 105). However a confirmatory semen analysis is still required for the diagnosis of infertility (74).

Inhibin B is produced by Sertoli cells andis responsible for the negative feedback regulation of FSH secretion in men (106). Decreased or even undetectable serum levels of Inhibin B and concurrent high levels of FSH have been reported in patients with disrupted spermatogenesis (107–110). Jensen et al. stated that the predictive power in detecting oligospermia, defined as semen concentration below 20 mill/mL, among men with a serum inhibin B below 80 pg/L and a serum FSH above 10 IU/L, was 100% (109). More recently a cutoff value of Inhibin B of 97.1 pg/ml between normospermia and oligospermia, with sensitivity and specificity of 79.2% and 72.7%, respectively, has been reported (111). Inhibin B to FSH ratio levels < 23.5 ng/U is associated to impaired fertility (79). However controversial study results have also been reported showing a poor specificity of FSH and inhibin B in determining spermatogenic capacity in adult male childhood cancer survivors (112). Therefore, although FSH, inhibin B and inhibin B to FSH ratio to some degree indicate spermatogenic capacity, direct evaluation by semen samples, which can be repeated during the follow-up, should be recommended for all patients interested in their fertility potential (107).

Serum testosterone (TST) and luteinising hormone (LH) level measurement are useful to assess testicular Leydig cell function. They remain within the normal range after different chemotherapy regimens, such as escalated BEACOPP, supporting the hypothesis that Leydig cells are more resistant to cytotoxic chemotherapy and are not associated with impaired spermatogenesis (110). TST serum levels below normal ranges indicate hypogonadism which is responsible for sexual dysfunction, fatigue and increased cardiovascular disease risk (113). Therefore, in the event of symptoms of hypogonadism, it is recommended to measure TST levels in order to guide hormonal replacement treatment and ameliorate quality of life.

Based on available study results, no recommendation can be given on the optimal time interval between completion of lymphoma treatment and first gonadal function assessment tests, frequency and duration of surveillance (100). Based on expert opinion, we suggest performing gonadal evaluation during the follow-up at the request of the patient or when paternity is desired, starting 12 months after treatment completion, and repeating abnormal tests annually thereafter.

### 3.6 How long after the end of cancer treatments can conception be considered safe?

For female lymphoma survivors no specific data are available in literature (6). Although there are some data from other malignancies suggesting a “safety window” of 6 months after chemotherapy (114), the current indications for female lymphoma survivors are to avoid pregnancy in the period of greatest risk of lymphoma recurrence, corresponding to the first two to three years, calculated from the end of treatment (16). An interval of at least 1 year after the end of chemotherapy is also recommended in order to reduce pregnancy complications, which seem to be higher in this population (55, 56, 62). However, preconception counseling and timing should be personalized in relation to the prognosis of the disease and the patient’s age and desire for parenthood.

For male lymphoma survivors, data from longitudinal, prospective cohort studies are awaited to provide further evidence on the potential risk of congenital abnormalities. The only guidelines currently available, issued by the European Society for Medical Oncology (ESMO), advocate the deferral of childbearing for at least 12 months after cancer therapy, as a Grade C recommendation based on level IV evidence (65, 115, 116).

Patients should be aware that fertility may be resumed a few months after the end of antiblastic treatment, especially for males. Considering the reasons set out in this paragraph, female patients should be informed to adopt efficacious contraception for the first two to three years of follow-up. Male patients should also be informed to employ efficacious contraception for the first 12 months since last anti-cancer treatment.

### 3.7 Assessment and treatment of the disorders of the sexual sphere after anti-tumor therapy

#### 3.7.1 To whom should we address the evaluation of disorders of the sexual sphere?

Cancer treatments have been shown to induce several effects on psychological and interpersonal conditions that can negatively impact sexual function and satisfaction. Data on the likelihood of specific sexual disorders seem to be related to pre-diagnosis function, patient response, and support from the treatment team as well as specific treatments employed and efforts to mitigate potential problems.

For hematologic cancers, sexual function might not be initially crucial for this group of patients as the malignancy is not directly linked to a sexual organ, although high-dose chemotherapy, total-body irradiation and stem cell transplantation can be significantly detrimental to patients’ body image, intimacy, and sexuality (117–120). Studies on sexual function in onco-hematologic patients contrast in their methodological approach, and the literature overall remains limited, although the few studies that document the existence of sexual disorders consider this topic of great interest.

In a study of NHL survivors at least 25% of the sample reported sexual problems (121), as compared to HL survivors with a prevalence range between 12% and 62.5% (122–124). Research on interventions for sexual problems following treatment for hematologic cancers is largely absent, and the clinical management generally follows the same recommendations made to women following breast and gynecologic cancers and to men following prostate cancer. True barriers still exist for patients, providers, and institutions to discuss and address sexual dysfunction during and after cancer treatment. Two approaches are discussed to address the potential barriers of time constraints, providers’ feelings of embarrassment and fear, and assumptions about patients’ level of interest in sexual health concerns or potential reactions to sexual health discussions. These approaches include the implementation of a paper-and-pencil screening of symptoms, a self-report survey during routine clinic visits, and a reference guide to starting the conversation about sexual health with patients and survivors of cancer based on established frameworks for discussing this topic (76). Paper-and-pencil screening tools are available to briefly and preliminarily assess sexual function in patients with cancer and help determine which patients may require further specialized assessment and intervention.

#### 3.7.2 What tools should be used to evaluate any alterations in the sexual sphere in the post-therapy phase?

In recent years many efforts have been made to expand the tools available for assessing sexual problems in patients with cancer to include measures other than specific organ function, and research studies on these tools continue to be underway. Two instruments have been established as the most widely used and easily accessible to providers to measure sexual function in research. These instruments are the Female Sexual Function Index and International Index of Erectile Function.

##### 3.7.2.1 The female sexual function index

The FSFI is a 19-item self-report measure originally developed to assess female sexual function in women of any age, including perimenopause and post-menopause, in the general population. The FSFI takes approximately 15-20 minutes to complete and assesses function “over the past 4 weeks” in the following specific domains relevant to female sexuality: desire, arousal, lubrication, orgasm, satisfaction, and pain (125, 126). Although the FSFI only takes a few minutes to complete, a recently developed shorter version, not yet validated with oncological patients, is the FSFI 6-item version which takes approximately 3 minutes to complete (127). For the FSFI-19, a maximum score of 36 is foreseen. Different cut-offs have been identified according to the cohorts in which the test was used; a score lower than 26.55 would seem to indicate subjectswith sexual dysfunction, who could benefit from a psychological approach and a specialist gynecological evaluation (128, 129).

##### 3.7.2.2 The international index of erectile function

The IIEF is a 15-item self-report measure developed to assess erectile function in men in the general population. The IIEF measures function “over the past 4 weeks” in the following domains relevant to male sexuality: erectile function, orgasm, desire, intercourse satisfaction, and overall satisfaction. However, shorter versions of the IIEF are also available. Of these shorter versions, the IIEF 5-item version (IIEF-5 also known as the Sexual Health Inventory for Men/HIM374) has also been validated and used in patients with and survivors of cancer (127, 130). Regarding the IIEF questionnaire, a score below 26 is indicative of male sexual dysfunction (1-10: severe erectile dysfunction; 11-16: moderate erectile dysfunction;17-25: mild erectile dysfunction) (127). Patients with this score may be referred for psychological and urological consultation.

## 4 Conclusions

FP and parenthood-planning remain among the most important quality of life issues for young female and male patients with NHL and HL. A large number of publications underscore the relevance of these topics and support the recommendation to offer fertility counseling before starting anti-neoplastic treatment and in the follow-up of these patients, including assessment of sexual function (55, 61, 62, 131).

Statements were elaborated according to a literature search and administered to a panel of experts belonging to Fondazione Italiana Linfomi (FIL) and Società Italiana della Riproduzione Umana (SIRU), using the Delphi methodology to reach a consensus.

Results confirm that a multidisciplinary approach including onco-hematology and human reproduction specialists is needed to offer patients and treating physicians updated guidelines to address these issues. Moreover, controversies still remain on specific topics such as the optimal age to offer ovarian and oocyte cryopreservation to female patients and the efficacy of GnRH analogues to prevent POI. Panelists underlined that sexual issues are rarely addressed during treatment and in the follow-up of patients affected by lymphomas, and that more awareness and adequate assessment is needed.

The shared statements contained in this paper should offer practical guidance to help onco-hematologists, human reproduction specialists and their patients to address fertility and sexual issues during the disease trajectory and obtain a thoroughly informed decision.

## Data availability statement

The original contributions presented in the study are included in the article/**Supplementary Material**. Further inquiries can be directed to the corresponding author.

## Author contributions

Conceptualization, CM, SV, ES. Writing— original draft preparation, CM, SV, ES, GDP, SP, FP. Validation, AG, GG, FM, GC, LM, FP. Supervision, FP. All authors have read and agreed to the published version of the manuscript.
